# A comprehensive quantitative comparison of myocardial motion in mice, rabbits and humans using phase contrast MRI

**DOI:** 10.1186/1532-429X-14-S1-P54

**Published:** 2012-02-01

**Authors:** Bernd A Jung, Katja E Odening, Erica Dall'Armellina, Daniela Föll, Jurgen E Schneider

**Affiliations:** 1 Dept. of Radiology, Medical Physics, University Medical Center, Freiburg, Germany; 2 Dept. of Cardiology, University Medical Center, Freiburg, Germany; 3 Dept. of Cardiovascular Medicine, University of Oxford, Oxford, UK

## Background

Genetically manipulated animals like mice or rabbits play an important role in the exploration of human cardiovascular diseases. It is therefore crucial to identify animal models that closely represent the human cardiac phenotype.

## Methods

Phase contrast MRI [1] was used to measure regional three-directional LV myocardial motion with high temporal resolution in mice (N=18), rabbits (N=8), and humans (N=20). Radial, long-axis, and rotational myocardial velocities were acquired in left ventricular basal, mid-ventricular, and apical short-axis locations (see Table 1 for scan parameters). Positive radial velocities indicate contraction, positive long-axis velocities motion from base to apex, positive in-plane rotation is defined as clockwise viewed from apex to base. Global (averaged over the entire segmentation mask) and regional (by partitioning the LV into 16 segments according to the 17-segment model by the AHA) motion patterns were analyzed. Peak velocities were determined as well as velocity-ratios between lateral and septal wall, anterior and inferior wall, and basal and apical segments as dimensionless parameter for a comparison between species.

**Table 1:** Scan parameters

	Mice	Rabbits	Humans
MR scanner [Tesla]	9.4	1.5	1.5
Number of subjects	18	8	20
Matrix size	128 × 128	160 × 98	256 × 96
Spatial resolution [mm]	0.2 × 0.2	1.0 × 1.2	1.3 × 2.6
Slice thickness [mm]	1	4	8
TE / TR [ms]	2.1 / 4.6	5.4 / 7.6	5.0 / 6.9
Temporal resolution [ms]	4.6	7.6	13.8
Venc in-plane [cm/s]	6	10	15
Venc through-plane [cm/s]	8	15	25
Bandwidth [Hz/pixel]	1150	650	650
Flip angle [°]	15	15	15
Averages	2	4	1
Respiration control	Yes	No	Yes

## Results

**Figure 1:**
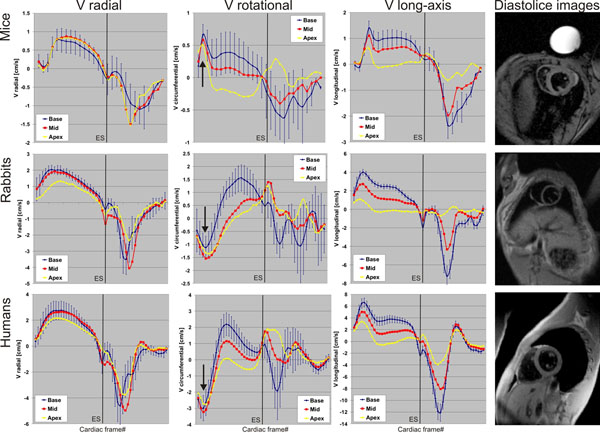


Similar behavior in the global LV myocardial motion pattern was observed in all species, particularly for the radial and long-axis motion as can be seen in Figure 1. However, a markedly different rotational behavior during early systole was found in mice, which exhibited clockwise rotation in all slice locations compared to counter-clockwise rotation in rabbits and humans (arrows in Figure 1). Regional analysis revealed significant differences in the velocity-ratio between lateral and septal radial and long-axis peak velocities between mice and rabbits (p<0.003), mice and humans (p<0.001), and rabbits and humans (p<0.04). Significant differences in the ratio between anterior and inferior wall were only found for long-axis velocities mainly between mice and rabbits/humans (p<0.02). The comparison of the ratio between basal and apical segments showed significant differences (p<0.001) for radial and long-axis velocities mainly for mice vs. rabbits and mice vs. humans.

## Conclusions

The results revealed a significantly different myocardial motion pattern in mice and rabbits compared to humans. Especially systolic rotation of rabbits more closely resembled human left ventricular performance, a finding that should be considered when investigating myocardial performance using mouse models. These findings underline that in the assessment of myocardial motion animal models cannot be conferred one-to-one to human beings. This deeper knowledge of mammalian-related differences in myocardial motion can help to select animal models that best mimic the human phenotype when investigating myocardial diseases.

## Funding

DFG FO 507/3-1.

## References

[B1] Jung JMRI 2006 24 1033

